# Beyond evidence: how actor dynamics and power shape knowledge translation for health policy in Kenya

**DOI:** 10.1093/heapol/czaf050

**Published:** 2025-07-31

**Authors:** Fatuma Hassan Guleid, Edwine Barasa, Gilbert Abotisem Abiiro, Jacinta Nzinga

**Affiliations:** Health Economics Research Unit, KEMRI-Wellcome Trust Research Programme, 2nd floor, 197 Lenana Place, Lenana Road, P.O Box 43640-00100 Nairobi, Kenya; Health Economics Research Unit, KEMRI-Wellcome Trust Research Programme, 2nd floor, 197 Lenana Place, Lenana Road, P.O Box 43640-00100 Nairobi, Kenya; Nuffield Department of Medicine, Center for Tropical Medicine and Global Health, New Richards Building, Old Road Campus, University of Oxford, Oxford OX3 7LG, United Kingdom; Department of Health Services, Policy, Planning, Management and Economics, School of Public Health, University for Development Studies, P.O Box TL1350 Tamale, Ghana; Health Economics Research Unit, KEMRI-Wellcome Trust Research Programme, 2nd floor, 197 Lenana Place, Lenana Road, P.O Box 43640-00100 Nairobi, Kenya; Liverpool School of Tropical Medicine, Pembroke Place Liverpool L3 5QA, United Kingdom

**Keywords:** knowledge translation, health policy, actors

## Abstract

Efforts to strengthen knowledge translation (KT) for policy-making often call for greater engagement with the policy process and its actors. Yet, existing KT approaches often focus on communication and dissemination of evidence and undertheorise the role and influence of policy actors on KT. As such, this study examines how, why, and to what effect policy actors shape KT. Our findings address a critical gap in the KT literature regarding the relational dimensions of KT for policy-making in low-middle-income countries. We utilised purposive and snowball sampling to identify participants who are involved in health policy-making and KT in Kenya. This included policy-makers, academics/researchers, knowledge intermediaries, and external partners (development and implementation partners). Data were collected through in-depth interviews (*n* = 32), observations (*n* = 52 h), and document reviews (*n* = 34). Data analysis was informed by a theoretical framework that combined perspectives from actor-centred institutionalism, Gaventa's PowerCube, boundary work, and coproduction. Our findings reveal how actor influence in KT is shaped by institutional mandates and roles, which, in turn, shape how actors perceive their position and authority in KT processes. While some actors viewed themselves as constrained to the role of evidence provision, others acted as boundary spanners across policy spaces, enabled by their institutional flexibility and financial resources. In addition, actor interests shaped when and how they exercised power to support or resist KT. Furthermore, access to policy spaces determined whose evidence was visible and perceived as legitimate, reflecting deeper power structures. These dynamics frame KT as a relational process mediated by political and institutional structures. As such, this study highlights the need to reconceptualise KT to integrate relational and structural dimensions, moving beyond evidence dissemination to addressing actor and power dynamics. It contributes novel insights into the interplay between actors, context, and power in shaping KT outcomes.

Key messages

- Actor roles and influence in knowledge translation (KT) is shaped by institutional mandates that affect their agency in KT-policy processes.
- KT is embedded in, and is constitutive of, policy-making: it reflects, maintains, or challenges institutional structures and power hierarchies.
- Efforts to improve KT should navigate institutional structures, enable boundary spanning roles, and shift power dynamics.

## Introduction

Knowledge translation (KT) has been framed as a predominantly technical practice to improve the accessibility, relevance, and application of research evidence during health practice and policy-making (Graham *et al*. 2006, Straus *et al*. 2009). However, studies on evidence use in policy-making suggest that these technical practices alone are often insufficient (Cairney and Oliver 2017, Cairney 2022). Instead, they suggest that KT in policy contexts is deeply relational and embedded in actor dynamics including actor interests, institutions, networks, and power (Trostle *et al*. 1999, Albert *et al*. 2007, Daniels and Lewin 2008, Allen *et al*. 2015, Barnsley *et al*. 2017, Huse *et al*. 2023). These studies highlight actors as dynamic individuals/groups with diverse values and varying degrees of power, all of which shape how they produce, frame, communicate, negotiate, and apply evidence (Nattrass 2008, Parkhurst 2012, Pelaez *et al*. 2013, Cairney and Oliver 2017, Parkhurst 2017, Biermann *et al*. 2020). While KT scholarship has evolved partly to recognize these factors, it still largely undertheorizes actors in terms of their situated and political roles (Bornbaum *et al*. 2015, Gagliardi *et al*. 2015, Kothari *et al*. 2017, Fafard and Hoffman 2020). This framing obscures how actors’ positions, agency, and institutional contexts shape KT and its outcomes (Head 2010, Murphy and Fafard 2012, Wiseman 2015).

Policy studies offer valuable insights for analysing actors in KT. For instance, actor-centred institutionalism (ACI) emphasizes how formal and informal rules, resources, and institutional structures shape actors’ choices and relationships in the policy space (Scharpf 2018). The advocacy coalition framework (Sabatier and Weible 2019) and policy network approaches further highlight how actors’ beliefs, networks, and access to policy-making spaces influence policy outcomes (Scott *et al*. 2014). These perspectives emphasize actor networks, beliefs, and power as relational and embedded, offering useful lenses for understanding how actors and their institutions constrain or enable KT.

Similarly, science and technology studies (STS) offer alternative conceptualizations of KT. STS pushes against the idea of a fixed, transportable body of evidence awaiting uptake, and instead emphasizes the situated, negotiated, and co-constructed nature of evidence. Concepts from STS suggest KT to be an active process of negotiation, displacement, and transformation of evidence by the actors involved (Callon 1984, Borst *et al*. 2022). For example, the concept of boundary work shows how actors actively construct or deconstruct distinctions between different types/sources of knowledge to maintain authority or legitimacy, revealing the role of actors in defining what is considered legitimate knowledge (Gieryn 1983, Hoppe 2010, Ødemark and Engebretsen 2022). Similarly, Jasanoff's notion of coproduction, different from coproduction in KT literature, challenges the assumption that evidence enters the policy process neutrally. Instead, it suggests that knowledge and policy are mutually shaped. From this perspective, who is seen as a credible actor, what counts as valid evidence, and how knowledge is acted on are all mutually constituted (Jasanoff 2004, Bandola-Gill *et al*. 2023).

These insights reveal blind spots in prevailing KT models about how actors are conceptualized and open new avenues for more reflexive and politically attuned KT strategies. They suggest that KT is a socially constructed and politically mediated actor-driven process that occurs through negotiation, contestation, and the exercise of power by actors with different institutional roles, knowledge claims, and authority. This view of actors is especially pertinent in low-middle-income countries (LMICs) where the dynamics of actor interactions and power imbalances differ significantly from other regions (Young 2005, Siregar *et al*. 2023, Ansah *et al*. 2024). For example, studies from LMICs’ policy processes have shown how external donors and development partners not only fund research but actively shape national health agendas by tying financial support to specific evidence priorities and programmatic goals (Dalglish *et al*. 2017, Gautier and Ridde 2017, Khan *et al*. 2018). Such dynamics can both enable KT and marginalize locally generated knowledge. This power asymmetry, coupled with inadequate institutional capacity, creates unique conditions that are rarely captured by KT models developed in high-income countries. The combination of limited empirical research on actor-driven dynamics in KT and the contextual uniqueness of LMICs presents a critical gap in the KT literature. Without a clear understanding of how different actors interact, exercise power, and influence KT in these contexts, the development of KT strategies risks being ineffective and perpetuating existing power imbalances.

This study asks: How do actors influence how KT happens in the health policy context and what are the outcomes of this influence? By examining these dynamics in an LMIC context, this study advances KT scholarship and informs contextually relevant design of KT.

For conceptual clarity, Box 1 lists the definitions and conceptualization of key terms used in this manuscript.

Box 1. **Definitions of key terms**

- Policy actors are defined as individuals/groups/institutions that influence the policy process.
- Policy is conceptualized as both substance (the content, design, and characteristics of a policy) and discourse (ideas and narratives shaping policy discussions).
- Policy-makers are defined as individuals within government institutions who are mandated to make binding decisions on health policies. They could fall into three categories: (i) mid-level or senior-level civil servants who develop, draft, and analyse policy options; (ii) high-level officials who hold ultimate authority to approve policies; and (iii) politicians involved in legislation.
- Evidence/knowledge is conceptualized broadly to include a wide range of sources, including but not limited to, research, experience, evaluations, surveillance, etc.

## Materials and methods

### Study design

This study used a cross-sectional qualitative research design. It was conducted in Kenya and focused on policy actors at the national and subnational levels.

### Study context

Kenya is an LMIC with a population of 47.5 million people. In 2013, governance of the health system was devolved; the national government is primarily responsible for developing health policies and regulations and overseeing healthcare at the tertiary level through the Ministry of Health (MoH). The county governments are responsible for the ownership, management, and delivery of health services including health workforce management (Masaba *et al*. 2020).

Significant reforms in Kenya's health system present a dynamic landscape for studying KT. The country is facing a triple threat of infectious diseases (Geteri *et al*. 2024), the rising burden of noncommunicable diseases (Mtike 2022), and expanding health inequities (Muinde and Prince 2023). A funding shortfall and a significant decrease in donor support exacerbate this (McDade *et al*. 2021). In response, the country is renewing its commitment to strengthening and scaling up its universal health coverage (UHC) initiatives. These high-profile national commitments have generated strong momentum for translating political will into policy and practice. Therefore, Kenya presents a microcosm of the challenges and opportunities of translating research evidence into policy that can offer valuable insights applicable in Kenya and other similar countries.

### Kenya's health policy process

**Figure 1.**
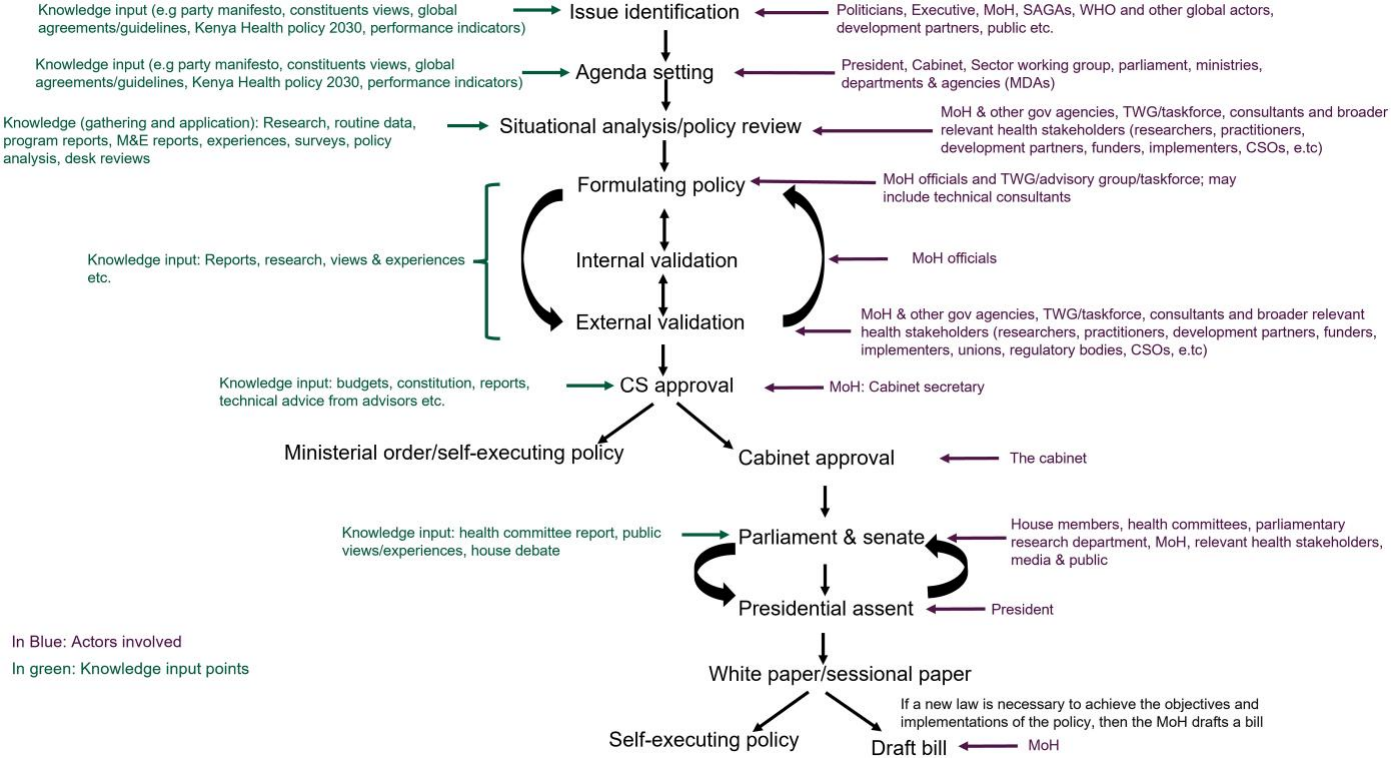
The policy-making process in Kenya. The chart shows which actors (nonexhaustive) are involved in each stage and key knowledge input points. MoH, Ministry of Health; SAGA, semiautonomous government agencies; WHO, World Health Organizations; CSO, civil society organiztions; TWG, technical working group; M&E, monitoring and evaluation.

Kenya's health policy-making process, on paper, appears straightforward with well-defined stages for evidence input (Fig. 1). The policy process can follow multiple pathways that yield different types of outputs (e.g. ministerial orders, legislation, guidelines, strategic plans, etc.). Each pathway involves different institutional procedures and actor configurations. The figure helps visualize which actors are involved in the policy process, at which point, and where opportunity for KT is likely or limited. For instance, the situational analysis and external validation processes allow for broader actor engagement and offer formal spaces for diverse input. In contrast, parliamentary debates, where access is restricted, narrow engagement.

While there is no explicit requirement for research use during policy-making, the MoH has established organizational structures and guidelines to support using research during policy-making. For instance, the Division of Research and Innovation, housed within the MoH, is mandated to make research more available and accessible for policy-making. However, the impact of this on research use is yet to be reported.

### Case selection

Rather than tracing a case(s), this study prioritized breadth over depth to explore how KT dynamics vary across a range of policy processes. Our aim was to capture variations in actor configurations, power dynamics, and evidence use depending on the type of policy issue at hand. For example, contestation, negotiation, and compromise play a key role in making policies around human resources for health (HRH) in Kenya due to the many actors involved and the decentralized nature of HRH policy-making. These include national and subnational policy-makers, implementation partners, health worker unions, regulatory bodies, and training institutions, all with different interests. In such cases, research evidence has limited influence compared with actor interests. On the other hand, in cases such as vaccine decision-making, where policy-making power is centralized and limited to a few actors such as national-level bureaucrats and external donors, research evidence plays a bigger role. This variation allows one to examine how KT dynamics vary across different kinds of policy processes with different actor configurations.

We aimed to include recent system-level reforms (3–5 years) with initial selection based on relevance to both national and subnational policy decisions. This included policies related to Kenya's UHC push [e.g. UHC policy, primary healthcare policies, health financing policies, HRH policies, facility improvement fund (FIF) policies, etc.] As the interviews progressed, we included other policies that were used as examples by the study participants. This included reproductive health policies, malaria control policies, and the community health strategy.

### Study population and participant selection

The study was conducted at the national level within the MoH and subnationally within three purposively selected County Departments of Health (CDoH). The rationale for including both national and county policy-makers lies in Kenya's devolved governance structure. Although the national government sets overarching policy directions, counties have authority over planning, budgeting, and implementation of health services. They can also initiate legislation. However, this autonomy can lead to tensions and contestation between the two levels of government. This interplay serves as a critical site for studying how power is negotiated and exercised across government levels; not only in terms of who is involved, but control over how agenda setting and implementation is asserted, resisted, or restructured.

The county selection was based on (i) the presence of recent and active policy reforms to reduce recall bias; (ii) demonstrable engagement with research and evidence use in policy-making; and (iii) their capacity to provide insights into different institutional structures. Each county represents different features that highlight the role of actor power, interest, and KT. For example, County A has had long-standing institutional partnerships with a major research institution and has developed structures that explicitly incorporate research into policy processes, making it a good site for examining institutionalized KT practices. County B has worked closely with development and implementation partners and researchers alike. They recently implemented UHC reforms in ways that reflect participatory approaches to governance and use of research evidence and advice. County C represents the more marginalized and resource-constrained setting. They had just completed the FIF legislation process (which was supported by development and implementation partners). These factors allowed us to capture diverse dynamics such as the influence of external partners and institutional structures.

The study participants included the MoH and CDoH policy-makers, health system researchers, intermediaries, and other key actors (Table 1).

**Table 1.** List of participants interviewed.

Category	Number
Academics/researchers	8
National and county policy-makers	17 (9 at national level and 8 at county level)
Development/implementation partners	3
Knowledge intermediaries	3
Semiautonomous governmental agencies	1
Total	32

A purposive and snowballing sampling approach was used to identify and select participants. Purposive sampling ensured that key informants with direct experience and knowledge relevant to KT and health policy-making were included. Snowball sampling was used complementarily to identify additional actors who were involved in or were influential within the KT and policy-making processes and may have been overlooked in our initial sampling.

### Data collection

Data were collected through interviews, observations, and document reviews:

- Semistructured interviews: In-depth interviews were conducted with 32 key informants from the actor groups mentioned above. We used a semistructured interview guide (Supplementary Table S1) focusing on participants’ identities, roles, experiences with KT, the power dynamics they navigated, and how actors influenced evidence use in policy decisions. The guide was developed based on the study's objectives and a review of KT literature. It was refined based on pilot testing with a small group of respondents to ensure that questions were clear and relevant. Interviews lasted 40 min on average and were conducted face-to-face and virtually between January and August 2024. The interviews were conducted in English, audio-recorded, and later transcribed verbatim.
- Observations: Nonparticipant observations were conducted at policy meetings and KT activities such as dissemination forums, policy dialogues, and stakeholder consultation forums (52 h; from January 2023 to August 2024). Detailed field notes were taken to capture the actors present, their interactions, the discourse around the policy issue, and how evidence was discussed (Supplementary Table S1). These observations provided context on how actors interact in real time, especially when such dynamics are difficult to capture through interviews.
- Document reviews: We reviewed relevant documents, including health system policies, strategies, meeting notes, policy briefs, and reports (*n* = 34). For the review, we focused on the actors involved in developing the policy documents or who were invited to meetings, the formal roles of actors, and how they are positioned within institutional structures (Supplementary Table S1).

## Data analysis

### Theoretical framework

This study draws on a set of conceptual lenses to interpret how KT is shaped by actors. Rather than using a framework *a priori*, we employed an interpretive approach allowing concepts from different fields to help us make sense of patterns that emerged inductively from the data.

Gaventa's PowerCube was applied to explore how power dynamics shape actor engagement and KT within policy processes (Gaventa 2020). The PowerCube provides a 3D view of power. One dimension recognizes that power can be exercised in different forms- ‘visible’, ‘hidden’ (where power is exercised behind the scenes), or ‘invisible’ (shaping beliefs, norms, etc). Another dimension refers to the spaces (‘closed’, ‘invited’, and ‘claimed’) where power is exercised. The final dimension (levels) examines how power operates at different decision-making levels including at the ‘household’, ‘local’, ‘national’, and ‘global’ level.

ACI provided a lens to investigate how actors and institutions interact to shape KT (Scharpf 2018). Its dual focus on institutional logics and agency highlighted how formal and informal rules underlie actors’ roles that are internalized as identities within the KT-policy space, shaping actors’ perceived influence and legitimacy within KT processes.

Boundary work (Gieryn 1983) is understood broadly as the practices through which actors negotiate boundaries between science and other forms of knowledge. While Gieryn's foundational work emphasized science/nonscience demarcation, boundary work is essentially about asserting and contesting epistemic authority. Here, we used it to understand how actors construct, blur, or defend boundaries across the KT-policy space. It helped us interpret how certain evidence claims were legitimized over others, how credibility was negotiated amongst actors occupying different institutional positions, and the contested nature of what counts as evidence in such political spaces. We amplify this concept by adding the idea of ‘boundary spanners’ (Tushman 1977). These are actors who can traverse across boundaries and connect domains.

Coproduction (Jasanoff 2004), particularly as articulated by Sheila Jasanoff, challenges KT models by arguing that knowledge and social order are produced together. Scientific facts and political institutions co-evolve; the way evidence is made credible is mutually constitutive of institutional norms, cultural narratives, and power relations. Thus, scientific claims are never purely technical; they carry implicit assumptions about authority, responsibility, and governance. Importantly, coproduction emphasizes how descriptions like ‘objectivity’, ‘evidence’, and ‘policy relevance’ are not given but constructed through practices of authority. This concept was used to move beyond the idea that evidence simply ‘informs’ policy, by showing how evidence and policy are mutually constituted.

### Analysis

We conducted a thematic analysis using an iterative multiphase process that happened alongside data collection. The analysis involved both inductive and deductive reasoning, using theory as an interpretive lens to enhance the explanatory depth of our findings and contribute to a conceptual understanding of KT-policy. The analytical process followed the following overlapping steps:

1. Data familiarization and initial coding: Analysis began during data collection. Interview transcripts were read several times to gain familiarity and identify initial impressions. Early codes were generated inductively, exploring emergent patterns such as ‘influencing policy’ and ‘institutional influences’ while remaining close to the data and capturing the participants own language around their experiences.
2. Generating initial codes: We then developed a coding framework using NVivo software. This initial framework included descriptive codes (e.g. power practices, influence on evidence use, institutional influences, etc.) drawn from the data. Coding at this stage was open and flexible with constant comparisons across transcripts and other data sources.
3. Engagement with theory and deductive refinement: As coding progressed, we applied the concepts described in the theoretical framework. These concepts helped us refine and expand our coding structure with more analytical codes (e.g. ‘navigating institutional structures’, ‘boundary spanning roles’ etc.) thereby integrating theory into the analysis.
4. Searching for and reviewing themes: We collated all codes into broader themes that we felt captured repeated patterns across the dataset. For example, codes such as ‘evidence legitimacy’ and ‘hybrid roles’ were grouped into a theme around ‘boundary work in KT’. These themes were reviewed against the full dataset and refined for internal consistency and conceptual clarity.
5. Defining and naming themes: The final themes represented higher-order analytic categories that draw on both empirical data and conceptual insights. Themes such as ‘actor roles, identities, and agency are shaped by institutional structures’ demonstrate the movement from descriptive coding to theoretical interpretations. This approach moved beyond descriptive accounts to theoretically informed explanations of how and why power dynamics shape KT, offering insights for contextually relevant KT strategies.

### Ethical considerations

Ethical approval for this study was obtained from the author's institute prior to the start of the study. Informed consent was obtained from all participants at the start of data collection, and all data were anonymised to protect the identity of the participants.

### Reflexivity statement

Our dual roles as researchers and knowledge translators position us uniquely within the processes we are studying, giving us an insider perspective on how KT works for policy. However, we also recognize that our positionality is not neutral. We are embedded within the structures we analyse. While this proximity to policy actors allows us insight into the dynamics of KT, it also shapes our interpretations of the findings, particularly regarding the power dynamics that influence KT and policy-making. In addition, while we contributed to KT, we did not always have the authority or power to be invited into the high-level policy meetings where key decisions are made. Our exclusion from these spaces mirrors the power dynamics we seek to study. This reality is especially significant given the focus of this paper on power relations in KT processes. Our position within these power hierarchies shapes our ability to observe policy-making fully, and it is essential to recognize how this may limit our capacity to fully capture or critique certain power dynamics at play. Therefore, in interpreting our findings, we remained reflexive about how our power (or lack thereof) may have influenced data collection and interpretation, particularly regarding whose voices were more accessible and whose perspectives might remain underrepresented in this analysis.

## Results

The health policy space in Kenya is characterized by a multitude of actors (Table 2), each wielding different identities, roles, and sources of power that shape KT. The roles actors adopt in KT were shaped not only by their institutional mandates but also by how they perceive their own influence within policy spaces.

**Table 2.** Health policy actors and their roles and influence in health policy-making.

Actors	Role (nonexhaustive)	Source of power and their influence (non-exhaustive)
Politicians and other elected officials	Develop and pass laws/policies Provide leadership and focus	Public mandate Legislative authority Budgetary control Set policy priorities/agenda Allocate resources Influence public opinion
Bureaucratic actors, e.g. the Executive, Committees, boards, etc.	Support policy development, including implementation Operationalize and implement policy/law passed by elected officials/politicians May allocate tasks, responsibilities and define competencies for policy development and implementation Monitor policies and track outputs or outcomes	Managerial authority Policy implementation Develop and implement policies Manage programmes Oversee resource allocation Provide technical advice to executive and legislature
Semi-autonomous government agencies (SAGAs)	Delivers specialized health services as a complement of the central and county government	Implement policies Provide specified services Provide technical advice to executive and legislature
Regulatory bodies & councils	Regulation and enforcement Can set or change scope of practice, training, licensure requirements, etc. Develop/adopt guidelines or standards Monitor safety and quality of health services and continued competence of professionals	Legal authority Regulatory power Set standards and enforce compliance Issue licenses and permits
Academia and research	Contribute and share research expertise concerning the policy problem Engagement and potential influence with other actors to support policy processes	Technical expertise Knowledge generation
Intermediaries/knowledge brokers	Facilitate the translation of research into policy Provide technical expertise and assistance in accessing, assessing, and use of evidence for policy Build capacity for evidence use	Technical expertise Knowledge generation
Donors and implementation and development partners/international nongovernmental organizations	Funding or in-kind support for policy processes and interventions May have funding for a specific programme/intervention therefore have vested interest in how policies are developed Engagement and potential influence with state actors to support policy-making and implementation	Financial resources Technical resources International influence Provide financial support and other resources for policy processes Influence policy directions through funding priorities Influence policy direction through funding for implementation Provide consultants that spearhead the policy process and collect evidence Build policy-makers and implementer's capacities Set global health standards
Unions	Negotiate contracts and agreements with government Engagement and potential influence with other actors to support policy processes	Collective bargaining power Ability to disrupt services Influence policies through protests/strikes Advocate for policies that improve health workforce conditions
Civil society organizations (CSO)	Advocate for specific policies or legislations Support and monitor implementation Monitoring and oversight of the government Engagement and potential influence with other actors to support policy processes	Public support Advocacy skills Expertise Community voice Raise public awareness Mobilize communities Provide services
Citizens/Public	Share or contribute lived experience of the problem, the policy interventions, the implementation or the evaluation of the policy effort, and any expected outcome Participate in policy processes through public participation	Public opinion Voter influence Raise awareness Hold policy-makers and politicians accountable Influence political debates through advocacy Shape public understanding
Media	Report on and communicate facts or perceptions of the policies and processes to the public	Public opinion Voter influence Raise awareness Hold policy-makers and politicians accountable Influence political debates Shape public understanding

### Actor identities, roles, and institutional contexts

Actors’ perceived influence in translating evidence into policy was tied to their professional and institutional identities. These identities shaped not only how actors positioned themselves in relation to KT processes, but also the extent to which they recognized, embraced, or limited their potential influence. Some actors adopted a narrow identity that constrained their view of how they influenced KT. These fixed perceptions were rooted in institutional mandates and sectoral norms about what their roles were. For example, participants who identified solely as evidence producers/providers exhibited a constrained identity centred on the role of evidence provision. This identity reflects a traditional and more passive role in the KT process and indicates a perceived lack of influence beyond evidence provision:

For us it's mostly just to present the evidence. Show that this is what the evidence is telling us would be the best direction to go and keep advocating for whatever position the evidence is showing. But in the end, you know, that's all we can do. You can't go and do much more than that. **Intermediary**

In contrast, actors with fluid or hybrid identities demonstrated greater ability to align evidence with political and institutional demands and strategically engage with the policy process. Such identities emerged from cross-sectoral career paths, formal multirole mandates, or sustained exposure to different policy-making spaces. For example, a researcher who became a policy-maker described how this new identity influenced their expectation of how research should engage with policy:

…working through this [policy] process I also learnt that policy is bigger than research….and so today, when I expect a research team walking in to provide evidence for my board or one of the committees I serve on, I expect to hear what you [the researcher] think and then what are the alternative scenarios to that, and what are the options we have. **National policy-maker**

Boundary work and ACI reveal how institutional roles both limit and expand what actors perceive as their legitimate role in the KT-policy process and how some actors navigate role boundaries. In contrast to actors with constrained identities, actors with hybrid identities could act as boundary spanners. Such actors were able to leverage their experiences in different roles to bridge institutional divides:

My experience working in Government has been very key…being able to work in both spaces at very senior levels, you understand how government does its business…it has given me an understanding of how to navigate the government's bureaucratic processes. **Intermediary**

They also engaged with diverse actor groups due to their multiple roles and were able to blur the boundaries between institutions. This facilitated more collaboration between actors through strengthened relationships and facilitated knowledge exchange.

Similarly, role blurring occurred when actors took on informal roles beyond their formal institutional mandates. Such role blurring was driven in part by informal relationships between actors and institutional gaps/resource constraints. For example, researchers sometimes took on the role of funders by financially supporting (through research grants) meetings or the launch of a policy document.

Institutional structures played a critical role in shaping these identities and roles. For instance, subnational policy-makers perceived policy-making as the domain of national-level policy-makers, often seeing their own identity and role as merely implementing decisions. This discrepancy arose from the formal constitutional framework that assigns policy-making authority to the national government:

I would really consider myself as an implementer, even the way health is structured in the country, county governments are really more of implementers, while the national government through the Ministry of Health is rather the one in charge of policy formulation…it's because of the way the constitution assigns our responsibilities.’ **County policy-maker**

In contrast, development and implementation partners’ diverse institutional mandates enabled multirole engagement with policy (e.g. evidence generation, agenda setting, policy formulation, and implementation support). Such institutional flexibility allowed them to take on multiple roles across KT and policy processes and enabled actors in these institutions to work across boundaries and between institutions. As such, they exercised more influence over KT processes by co-producing the epistemic and institutional frameworks within which policy problems were defined and addressed.

Altogether, these findings suggest that actors’ ability to influence KT was shaped by the institutional structures within which they operated, thereby mediating their agency within the KT-policy process.

### Power dynamics shaping knowledge translation

**Figure 2.**
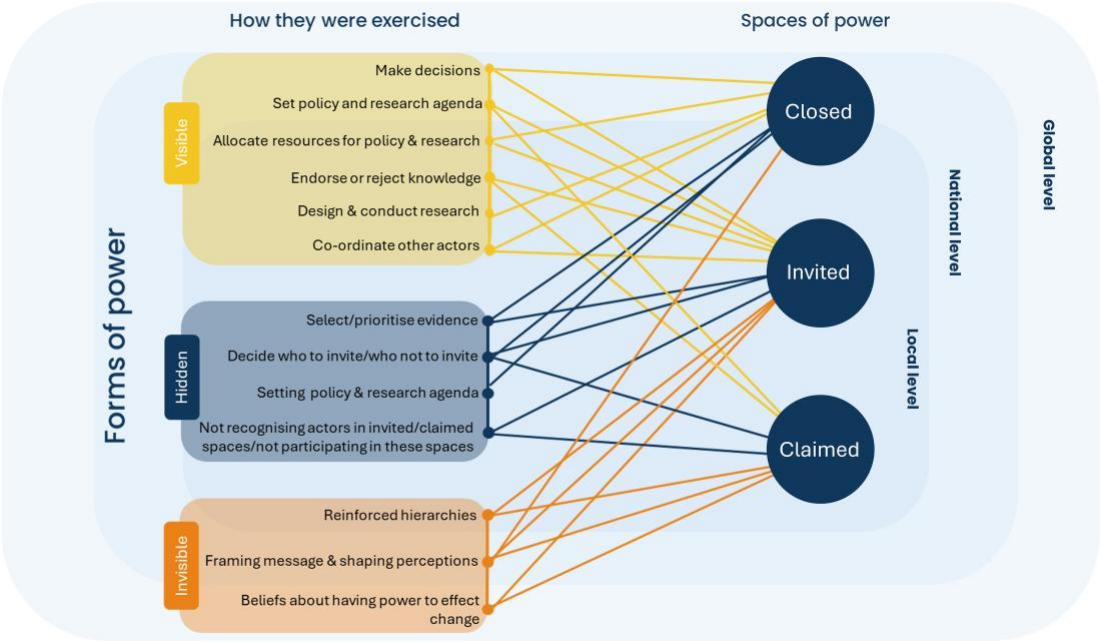
Schematic representing how various forms of power are exercised in the different policy spaces (nonexhaustive).

Actors’ identities and roles and the institutional structures that shape them mediate power dynamics that influence KT within policy-making. For example, within government institutions, power was exercised in hidden ways through selective consultations amongst a privileged few (closed spaces), where critical decisions were made before broader discussions occurred (Fig. 2). These practices highlight the hierarchies within government spaces, where certain actors wield greater influence based on their roles and institutional authority:

So, you have a board meeting, but another meeting happened last night, and decisions were made. And by the time you are coming to the meeting, there's no discussion taking place, you just see people voting for an agenda that has not been explored or there were no board papers. **National policy-maker**

The subsequent exercise of power in visible ways, such as formal ratification processes, served to legitimize these predetermined decisions, while maintaining an appearance of transparency. Similarly, external development and implementation partners exercised power both visibly and in hidden ways. They not only funded policy meetings and gathered evidence but also significantly influenced agenda-setting and resource allocation for implementation. While they exercise power in hidden ways through their control over agenda-setting and expertise, their roles are legitimized through mechanisms such as formal participation in policy dialogues and in advisory roles.

Furthermore, the decentralization of governance in Kenya granted local governments the authority to formulate and implement local policies. However, this redistribution of power is tempered by national governments retaining control over policy-making authority and resource allocation. This tension reflects the different institutional logics and power at play. For example, in formulating and implementing county-specific FIF laws, counties took the lead. However, the national government (through the MoH) sought to standardize their implementation by developing a model FIF law. When counties failed to implement this model law, the MoH moved to parliament to create legislation that counties would have to adhere to. Nevertheless, county governments exercised power in hidden ways through nonendorsement or nonimplementation of national policies, reflecting the ongoing negotiation of authority between governance levels:

They (county governments) have a very strong say now. I would say that it's increasingly becoming stronger that if they say no to something, people have to think twice. **Researcher**

The interplay of power within the government and the asymmetries maintained by hierarchical decision-making was also seen in the way the government strategically employs consultation and negotiation to secure stakeholder buy-in in invited spaces. While these spaces are presented as participatory, their creation and functioning expose layers of visible, invisible, and hidden exercises of power. First, establishing invited spaces signalled power being exercised both visibly and in hidden ways by defining who is allowed to participate, when, and under what terms. Power exercised invisibly shaped these spaces by structuring discussions to align with dominant narratives, such as governmental or donor-driven agendas. For instance, policy-makers employed extensive consultation when developing HRH policies to secure buy-in, subtly guiding outcomes towards predetermined objectives:

Nowadays, from the outset, involving as many of the relevant actors in policy development is critical. One, making sure that all potential areas are looked at and then secondly to make sure that there is some buy-in to that policy across the stakeholders and when it goes for implementation, once you have a buy-in, these people will be able to work towards that common objective that you have set for yourself. **National policy-maker**

External development and implementation partners complicate these dynamics by leveraging their resource dominance and perceived expertise to assert influence within these invited spaces. By shaping the agenda, they perpetuated their ideologies, sometimes at odds with local contexts or priorities especially in policies around culturally sensitive issues such as reproductive health:

These policies that are partner driven.. like for example the X policy took long, at one point our former minister signed it but it was returned because of an interest. There was a partner who was really pushing for [intervention] to be included. **National policy-maker**

This power imbalance is exacerbated by resource constraints for policy-making activities, which limited broader consultations and increased reliance on external funding. As such, power was exercised visibly as a performative tool, signalling inclusivity, while hidden and invisible forms of power orchestrated who holds real influence in these discussions:

One of the things I’ve noticed is that you will have maybe three or four people from Ministry of Health and 20 people from other organizations. Many of the large non-governmental technical organizations or people like [development partner] or other donor partners. And the weight of power in the room is not with the government. **Researcher**

Local actors, however, strategically resisted these pressures, by leveraging multistakeholder consultations as a tool for resistance. Through collective resistance, they could reassert local priorities, showcasing how less powerful actors can employ relational and technical expertise to navigate power imbalances as was reported during the formulation of reproductive health policies:

There was a partner who was really pushing for [intervention] to be included in the policy… Eventually it was not included. The people [other local actors] fought it hard, and it was not included. **National policy-maker**

On the other hand, researchers and intermediaries, in their efforts to facilitate KT, create both invited and claimed spaces through policy dialogues and dissemination activities to create their own platforms of influence:

What was reasonably powerful was making sure we had all stakeholders, including the people who were actually nurses who were delivering care. Because bringing those different voices together slowly changed some of the, not all, but some of the attitudes of the senior nursing policy makers and people in Ministry of Health. **Researcher**

However, these spaces often struggled to attract high-level policy-makers, whose participation is shaped by broader power dynamics. The lack of engagement reflects the invisible exercise of power by shaping perceptions about the relevance and legitimacy of these spaces.

Actor interests help explain how and why actors exercised power. Drawing from ACI, actors are not only embedded in institutions but are also strategic agents whose behaviour is shaped by what they stand to gain. Our analysis showed that actors with strong interests in a particular policy direction, such as the national government seeking to reform social health insurance, were more likely to exert influence in the policy process:

In this dispensation [health financing reforms], we are seeing a lot of influence from the President's office……… I don't think the current reforms are necessarily based on evidence. **Development partner**

Interests also influenced how power was exercised. For some, such as development partners, power was exercised visibly by funding policy development activities:

If they have an interest in the area, they will also fund that area of formulation. Basically, the concept notes and all that, data collection and adoption and some towards the implementation side. **National policy-maker**

For others, especially those without formal authority, power was exercised through relationships, persuasion, or withholding support.

### Influence of actors on knowledge translation

The influence of actor identities, roles, and power dynamics on KT is a critical determinant of how evidence is disseminated, interpreted, and ultimately used in policy-making. The findings reveal that power asymmetries, coupled with entrenched actor identities and roles, lead to significant influences on KT:

#### Boundary spanning

The analysis showed that actors, particularly development and implementation partners, operated across multiple stages of the policy process. These actors played what can be described as ‘boundary-spanning’ roles, leveraging their institutional resources, mandates, and technical expertise to influence both the generation and the uptake of evidence. Their capacity to blur and connect distinct boundaries (e.g. research and policy, national and county levels) enabled them to participate in the construction of knowledge and policy across governance levels, deciding which evidence is considered and how it is interpretated and used.

#### Selective use of evidence

At the legislative and executive levels, political priorities and power dynamics heavily influenced how evidence was engaged with, often sidelining KT processes. Evidence that conflicted with political goals or challenged policy-makers’ reputation or performance was disregarded, while time pressures driven by political expediency where decisions were made hastily left limited time for evidence-informed deliberation. This was particularly evident in politicized issues such as social health insurance reforms:

There was no time to inform members, you see?……there was no time, and they didn't even want to hear because their party wants this thing passed as fast as yesterday so there was no time to advise appropriately so it really affects [research use]. **Researcher**

Evidence was also strategically reframed to suit political agendas, such as using data on poor health facility performance to justify building new facilities for political gain rather than addressing systemic issues. These findings underscore how political agendas limit the scope and influence of KT in these high-level policy-making spaces.

#### Privileging of external knowledge and resource control

Development and implementation partners significantly influenced KT processes due to their resources and the privilege accorded to their knowledge. Their financial contributions and institutional mandates often allowed them to set agendas, determine which evidence is produced, and shape policy priorities, creating a dynamic where local actors felt compelled to align with donor interests rather than local needs:

So, they can in be influential because they can bring in funding and, you know, Ministry of Health is very constrained. So, sometimes they will take up an idea because it's coming attached to funding which they need. **Intermediary**

This privileging of external knowledge, often presented as definitive or indispensable, can marginalize alternative perspectives, and limit the demand for new evidence or locally relevant evidence:

Well, so, they can make it seem that new knowledge is not needed. They can make it seem that, you know, “here's the money, we’ve been told the answer, here's the money, lets do something” rather than, actually, is this the right thing to do? or does it work?. **Researcher**

Moreover, when these partners withdrew funding or failed to invest in critical stages of the policy process, their absence stalled KT and policy progress, highlighting their influence in these processes:

If the partner pulls out, for example you find maybe the Government is not able to come in to complete that process and that has happened in a number of [policy] documents…. When they pull out, it stalls. When another partner comes in, it picks up. **National policy-maker**

These findings suggest that these actors gained influence because the policy process itself was shaped in ways that co-produced their authority by linking resources, evidence, and decision-making in mutually reinforcing ways.

## Discussion

This study set out to examine how actors, through the roles they play, their power dynamics, and institutions, shape the way KT processes unfold in policy settings. We adopted an interpretive approach to the analysis drawing on concepts such as coproduction, boundary work, actor-centred institutionalism, and the PowerCube to understand these various influences across different policies and governance levels.

A key finding from this study is that actors’ influence in the KT process is closely tied to how they internalize their institutional roles, which, in turn, shape their self-perceived identities and ability to influence policy through KT. These identities were not fixed but formed through the interaction of actors with institutional structures, norms, and expectations. ACI emphasizes that institutions structure the ‘playing field’ defining what is expected, but actors retain agency to act strategically within those constraints (Scharpf 2018). We found that actors in this study exhibited varying degrees of agency based on how their institutional roles framed their purpose in the KT-policy process. For example, actors with constrained identities reflect how institutional norms limited the possibilities for agency even when such actors were involved in policy spaces. In contrast, some actors displayed hybrid identities, often from inhabiting hybrid roles, and participating across multiple stages of the policy process (from evidence generation to implementation). An example of this is development and implementation partners who had greater latitude to act across policy spaces. Their ability to navigate and blur institutional and epistemic boundaries allowed them to function as boundary spanners. This type of boundary work was power-laden: by financing and framing policy agendas, they redefined the ‘rules of the game’ positioning themselves as legitimate brokers between knowledge production, exchange, and policymaking. These actors used this position to co-construct both policy problems and the evidence used to address them. Our findings also suggest that KT processes are inseparable from the production of institutional authority. It highlights how actor interests, roles, and institutional mandates are intertwined with the constitution of evidence, its legitimacy and the organization of KT-policy systems. These findings imply that KT influence is not simply about position or access but about how institutional arrangements shape actors’ self-perception, scope of action, and ability to navigate power. For KT practice, this means structuring institutions in a way that enables actors such as researchers to engage more effectively with policy spaces and enable them to play boundary spanning roles, for example, by affording opportunities such as formal secondment in policy institutions and creating incentives for engaging actively with policy-making (Jenkins and Anstey 2017).

Central to the study findings is the layered exercise of power, with visible, hidden, and invisible forms of power shaping who participates in policy spaces, whose knowledge is valued, and what decisions are made. While institutional mandates shaped the formal spaces actors occupy and the actions they take, actor interests influenced how and when these actors exercised power in KT processes. The interplay of interests here is multifaceted: policy-makers may prioritize public interests rhetorically, while advancing personal interests (such as political advancement). Similarly, development partners advance their interests overtly in promoting policy directions by funding policy meetings and implementation costs. Crucially, these interests influence the intensity and form of power exercised. These findings align with well-established insights from policy studies, particularly regarding the influence of power dynamics, institutional norms, and actor roles on policy-making and evidence use (Oliver *et al*. 2014, Cairney and Oliver 2017, Parkhurst 2017). However, this study explicitly links these dynamics to KT processes and outcomes, particularly in the health policy domain.

Policy-makers’ selective use of evidence can undermine democratic accountability. In addition, the privileging of external actors’ knowledge aligns with critiques in global health governance about the entrenchment of external ideologies and marginalization of local evidence (Ong’era *et al*. 2021). However, this study extends these discussions by illustrating how such dynamics inhibit the uptake of context-specific evidence, undermining the relevance and utility of KT efforts. These findings also point to the need to consider power asymmetries and how structural inequities rooted in resource disparities and global institutional hierarchies reproduce exclusionary KT-policy ecosystems (Oliver and Pearce 2017). Advancing equitable KT requires surfacing of these interests and how they shape KT. Institutional safeguards that mitigate the monopolization of KT by powerful actors would be necessary. Ultimately, these findings highlight KT as a contest of interests, where the exercise of power shapes whose interests prevail, and where outcomes of these contests can also reinforce or transform existing power relations. By linking the power dynamics inherent in policy-making to the form and extent of KT practice, this study highlights the need for KT approaches that go beyond evidence dissemination to actively address power asymmetries.

The strength of this study lies in its multilayered analysis of how actors shape the KT processes, providing valuable insights for KT strategies. However, our decision to focus on broad health systems policies rather than specific policies may have impacted the findings. While this approach provided breadth, allowing for the examination of different processes, it limited depth, potentially missing key actors and nuances within specific health policies. In addition, the potential underrepresentation of marginalized perspectives is a notable limitation, as they restrict a comprehensive understanding of broader influences shaping KT outcomes.

## Conclusion

By drawing on theoretical concepts from ACI, boundary work, the PowerCube, and coproduction, this study illustrates that KT is not merely a technical process of transferring evidence into policy. Rather, it is a politically situated practice shaped by institutional structures and actor interests and produces norms of legitimacy and authority. These lenses enable a richer understanding of the complex actor dynamics that influence whether, how, and why evidence is taken up in policy. It offers insights for designing KT strategies that are more politically and institutionally attuned in similar LMIC settings with devolved governance.

## Supplementary Material

czaf050_Supplementary_Data

## Data Availability

The data underlying this article cannot be shared publicly for the privacy of individuals that participated in the study. De-identified excerpts of the transcripts relevant to the study can be available upon reasonable request to the corresponding author.

## Reflexivity statement

This paper reflects inclusivity in authorship, comprising a team of two women and two men, all based in low- and middle-income countries (LMICs), aligning with the study's focus on LMIC contexts. The authorship includes a PhD student as the lead author and three senior researchers with varying levels of seniority and experience, ensuring a balance of perspectives across gender, career stage, and regional expertise.
