# Supplementary material for: Beyond evidence: how actor dynamics and power shape knowledge translation for health policy in Kenya
Source: Health Policy Plan. 2025 Jul 31;40(8):819–30. doi: 10.1093/heapol/czaf050 (PMC12448805; doi:10.1093/heapol/czaf050)
Supplement: czaf050_Supplementary_Data [file czaf050_supplementary_data.docx]

Supplementary table 1: Data collection guides

| Data collection method | Guide |
| --- | --- |
| In depth interviews questions | 1. Could you briefly describe your professional background?   *Prompts: Work experience, education experience?*   1. Can you describe your formal role in (institution, specific policy/ policy space) or affiliations? 2. For non-policy-makers: Are you a part of any policy groups e.g technical working groups, advisory groups etc.?   Prompt: *Can you describe your role in those spaces? How did you get involved? How long have you been in this policy groups?*   1. What was your role in the development of [ policy]   *Prompt: How did you get involved?*   1. Could you describe the other actors you have interacted with/who were involved in that policy space?   Prompts: *What are their roles/what roles have they played in this policy process? How do they approach the policy issue? How did they get involved? How frequently/ to what extent do you work with these actors during the policy process/in the policy space?*   1. Do you collaborate/build relationships with these actors? Prompts: How are perspectives/interests amongst actors shared and navigated? What factors facilitate/hinder the development of these collaborations/relationships? 2. In your opinion, did you feel that these other actors had an impact on how research knowledge was generated and used in this policy space? How? |
| Non-participant observations checklist | 1. What is the goal of the meeting/engagement? 2. Who is present? What formal roles/affiliations do they hold? 3. Who initiated the engagement? 4. Who is funding it? 5. Where is the engagement taking place? 6. What is the tone of interaction (collaborative, neutral etc) 7. Is there alignment/conflict between actors 8. Who is dominating/leading the discussions? 9. What evidence/arguments are being presented/emphasised? 10. Are decisions being made? Which? How? 11. Is evidence used to inform the decision? Which type? How? |
| Document review checklist | 1. What is the type of document? 2. Who authored/what institution is it affiliated with 3. What is the stated purpose of the document? 4. Who are the key actors mentioned in the document? 5. What are their roles? 6. What types of evidence are included? |
